# The proofreading activity of Pfprex from *Plasmodium falciparum* can prevent mutagenesis of the apicoplast genome by oxidized nucleotides

**DOI:** 10.1038/s41598-020-67853-2

**Published:** 2020-07-07

**Authors:** Minakshi Sharma, Naveen Narayanan, Deepak T. Nair

**Affiliations:** 1 Regional Centre for Biotechnology, 3rd Milestone, Faridabad-Gurgaon Expressway, Faridabad, Haryana 121001 India; 2 Kalinga Institute of Industrial Technology, Patia, Bhubaneshwar, Odisha 751024 India; 3 Manipal Academy of Higher Education, Madhav Nagar, Manipal, 576104 India

**Keywords:** Biochemistry, Molecular biology, DNA replication

## Abstract

The DNA polymerase module of the Pfprex enzyme (PfpPol) is responsible for duplication of the genome of the apicoplast organelle in the malaria parasite. We show that PfpPol can misincorporate oxidized nucleotides such as 8oxodGTP opposite dA. This event gives rise to transversion mutations that are known to lead to adverse physiological outcomes. The apicoplast genome is particularly vulnerable to the harmful effects of 8oxodGTP due to very high AT content (~ 87%). We show that the proofreading activity of PfpPol has the unique ability to remove the oxidized nucleotide from the primer terminus. Due to this property, the proofreading domain of PfpPol is able to prevent mutagenesis of the AT-rich apicoplast genome and neutralize the deleterious genotoxic effects of ROS generated in the apicoplast due to normal metabolic processes. The proofreading activity of the Pfprex enzyme may, therefore, represent an attractive target for therapeutic intervention. Also, a survey of DNA repair pathways shows that the observed property of Pfprex constitutes a novel form of dynamic error correction wherein the repair of promutagenic damaged nucleotides is concomitant with DNA replication.

## Introduction

Reactive Oxygen Species (ROS) arise in the cell due to impaired respiration or as natural byproducts of metabolic pathways. ROS reacts with different biomolecules and can impair their function and thus have an adverse effect on cellular physiology. ROS are known to oxidize the nucleotide pool. Among the dNTPs, dGTP is especially vulnerable to oxidation and 8-Oxo-2′-deoxyguanosine-5′-triphosphate (8oxodGTP) represents the most frequently occurring damaged nucleotide^1,2^. 8oxodGTP is frequently misincorporated opposite template dA in genomic DNA during replication and this event can lead to deleterious transversion mutations. 8oxodGTP misincorporation is known to be responsible for adverse physiological outcomes such as age-related degenerative disorders, cell death, or cancer^3–6^. To avoid the transversion mutations, 8oxodGMP present in DNA is repaired by multiple redundant pathways^7,8^.

ROS are naturally generated in organelles that are sites of metabolic processes such as the apicoplast in *Plasmodium falciparum*^9,10^. The apicoplast is an unusual plastid-like organelle essential for the survival of the malaria parasite. This organelle is the site of many important metabolic processes such as fatty acid synthesis, iron-sulfur cluster, isoprenoid and haem biosynthesis^9,10^. The apicoplast harbors a circular genome (~ 35 kb), which is replicated by the enzyme named Pfprex (*Plasmodium falciparum* plastidic replication/repair enzyme complex). Pfprex polypeptide harbors polymerase, primase and helicase activities and is centrally involved in the replication of the apicoplast genome by a bidirectional theta mechanism^11–15^. The *pfprex* gene is encoded in the nucleus, and the polypeptide is transported into the apicoplast due to a bipartite leader sequence (BLS) at the N-terminus^12,13^. Within the apicoplast, the Pfprex polypeptide fragments and the polymerase module separates from the primase-helicase activities^13,14^. The DNA polymerase activity of Pfprex is the primary enzyme responsible for duplication of the genome of this organelle^14,15^. Pfprex plays an important role in the survival of the parasite as any defect in the replication of the apicoplast genome results in the death of the organism^14,16,17^. Also, since Pfprex is of bacterial origin having no known orthologues in humans, this enzyme is an attractive target for the generation of novel antimalarial therapeutics^11,14^.

The polymerase module of Pfprex (named PfpPol) belongs to the A-family of polymerases and possesses 5′-3′ polymerase and 3′-5′ exonuclease or proofreading activity^14,15,18^. The PfpPol enzyme exhibits high fidelity of DNA synthesis^14^ with reported error rates ranging from 10^−4^ to < 10^−6^. It is the only known DNA polymerase in the apicoplast^15,18^ and, therefore, is more likely to encounter 8oxodGTP in the ROS-rich environment of the apicoplast, which can adversely affect replication. The majority of the A-family polymerases prefer to insert incoming 8oxodGTP opposite template dA except DNA polymerase I (*E. coli)*, which incorporates the oxidized nucleotide opposite both dA and dC^19–23^. The oxidized base of 8oxodGTP tends to adopt a syn conformation to prevent steric repulsion between the oxygen atom at the 8 position and the triphosphate moiety. As a result, it prefers to form an 8oxodGTP(syn): dA(anti) Hoogsteen base pair, which has the same C1′-C1′ distance as a canonical dTTP: dA Watson–Crick base pair^24–29^. Consequently, the 8oxodGTP(syn): dA(anti) Hoogsteen base pair can be accommodated in the active site of DNA polymerases (dPols) without any distortion of DNA or enzyme structure and consequently a number of dPols tend to misincorporate 8oxodGTP opposite dA^24,30^.

There was no information available regarding the activity of PfpPol with respect to oxidized nucleotides such as 8oxodGTP. Our studies showed that PfpPol is able to incorporate 8oxodGTP into the growing end of the primer and there is no impediment to replication following the insertion of the oxidized nucleotide. Since the incorporation is opposite adenine in the template and is not desirable, the exonuclease activity of PfpPol was checked to remove the misincorporated 8oxodGTP. The exonuclease activity was successful in removing the damaged base and the amino acid residues responsible for this heightened exonuclease activity were identified.

## Results

### Primer extension assays

**Figure 1:**
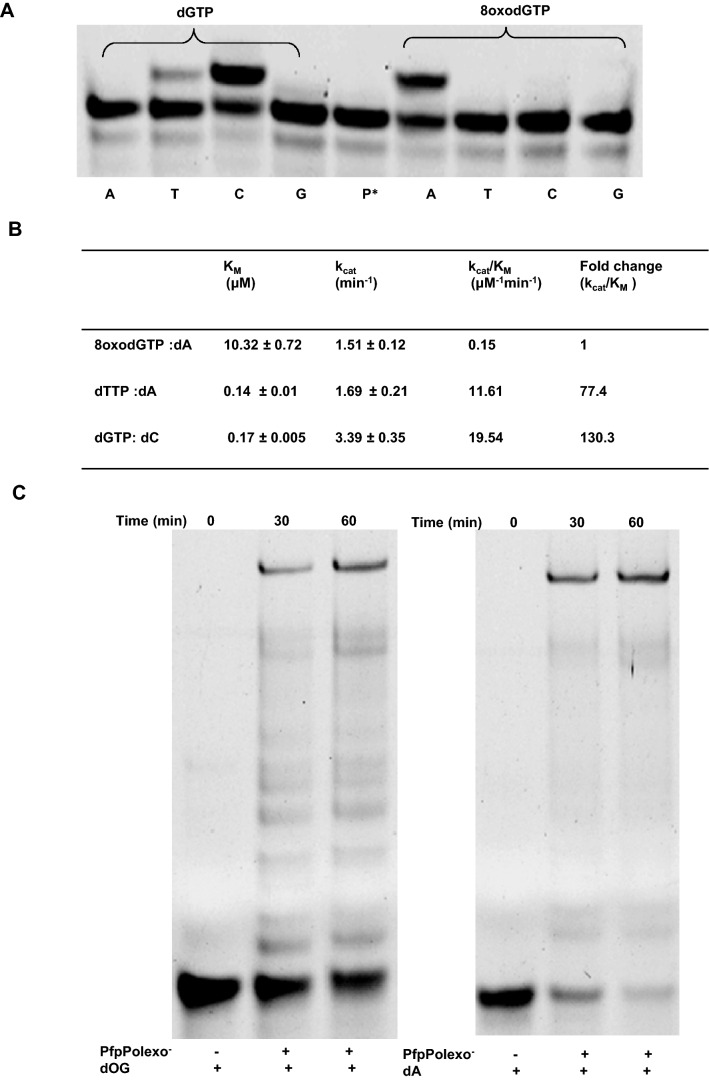
Activity of Pfprex *vis-a-vis* 8oxodGTP. (**A**) The ability of PfpPolexo^−^ to incorporate dGTP and 8oxodGTP opposite all four template nucleotides was compared. 30 nM PfpPolexo^−^ was incubated for 30 min with 100 nM of 5′ FAM labeled DNA and 5 µM of either dGTP or 8oxodGTP. P* represents only DNA. A, T, C and G denote templates dA, dT, dC and dG, respectively. It was observed that PfpPolexo^−^ incorporates 8oxodGTP opposite dA and not dC. (**B**) Kinetics parameters are displayed for the incorporation of 8oxodGTP opposite dA, dTTP opposite dA and dGTP opposite dC by PfpPolexo^−^. (**C**) PfpPolexo^−^ can extend from 8oxodGMP present at the primer terminus to the same extent as from dA and, therefore, the presence of the oxidized nucleotide at the primer terminus does not impede DNA synthesis.

The wild type (wt) PfpPol and a variant lacking exonuclease activity (PfpPolexo^−^) were purified to high homogeneity (Fig. S1B). The identity of the purified proteins was confirmed using peptide fingerprinting of Mass Spectrometry. PfpPolexo^−^ was assessed for its ability to incorporate the oxidized nucleotide 8oxodGTP into DNA. Primer extension assays show that PfpPolexo^−^ incorporates 8oxodGTP opposite dA and not dC in the template (Fig. 1A). The observed catalytic efficiency of incorporation of 8oxodGTP opposite dA is 0.15 μM^−1^ min^−1^. In comparison, the catalytic efficiencies for the addition of dGTP opposite dC and dTTP opposite dA are 19.54 and 11.61 μM^−1^ min^−1^, respectively (Fig. 1B). Therefore, the catalytic efficiency of incorporation of 8oxodGTP opposite dA is only 130- and 77-fold lesser than that for dGTP opposite dC and dTTP opposite dA, respectively (Fig. 1B). The frequency of incorporation of 8oxodGTP opposite dA in comparison to the addition of dTTP opposite dA (0.013) is nearly threefold higher than that for the error-prone DNA polymerase IV from *E. coli* (0.005)^24^. These experiments show that PfpPol has substantial ability to misincorporate 8oxodGTP opposite dA and therefore, in the presence of ROS, there is a possibility of one 8oxodGTP incorporation opposite every 100 dA nucleotides in the AT-rich genome (35 kb) of the apicoplast of *Plasmodium falciparum*.

### Extension post misincorporation of 8oxodGTP

The human telomerase has been shown to incorporate 8oxodGTP but cannot efficiently extend the 8oxodGMP terminated primer causing chain termination^31^. To check whether PfpPolexo^−^ stalls or continues replication after adding 8oxodGTP to the growing primer chain, we used DNA substrate (dOG) terminating with 8oxodGMP in the primer strand (Table 1). On addition of all dNTPs, this primer was fully extended by PfpPolexo^−^ (Fig. 1C). Hence, as seen for Polγ (A-family) from humans, Klenow fragment (KF) from DNA polymerase I (A-family) from *E. coli* and Polα (B-family) from *Bos taurus*, the presence of 8oxodGMP in the primer at the 3′ end does not impede replication by the PfpPol enzyme^21,22^. Due to the high frequency of incorporation of 8oxodGTP opposite dA by the polymerase activity (PfpPolexo^−^) and its ability to extend from 8oxodGMP present opposite dA, the 8oxodGMP: dA mismatches can appear recurrently in the apicoplast genome. This raises the possibility that the exonuclease domain may remove such mismatches and thus prevent the appearance of transversion mutations that may adversely affect biochemical pathways that operate in the apicoplast.

**Table 1:** List of DNA substrates.

**Templates**	
1	A_T_: 5*′* TCCTACCGTGCCTACCTGAACAGCTGGTCTCGCTAATGCCTACGAGTACG 3*′*
2	T_T_: 5*′* TCCTACCGTGCCTACCTGAACAGCTGGTCACACATATGCCTACGAGTACG 3*′*
3	C_T_: 5*′* TCCTACCGTGCCTACCTGAACAGCTGGTCATAGTCATGCCTACGAGTACG 3*′*
4	G_T_: 5*′* TCCTACCGTGCCTACCTGAACAGCTGGTCACATAGATGCCTACGAGTACG 3*′*
**Primers**	
5	P15: FAM 5′ CGTACTCGTAGGCAT 3*′*
6	POG: FAM 5′ CGTACTCGTAGGC_s_A_s_T*X* 3′ *X* = *8oxodGMP*
7	PG’: FAM 5′ CGTACTCGTAGGC_s_A_s_T*G* 3*′*
**Template- primer DNA substrates:**	
8	dG: 5*′* TCCTACCGTGCCTACCTGAACAGCTGGTCACATAGATGCCTACGAGTACG 3*′*
	3*′* TACGGATGCTCATGC 5*′*
9	dT: 5*′* TCCTACCGTGCCTACCTGAACAGCTGGTCACACATATGCCTACGAGTACG 3*′*
	3*′* TACGGATGCTCATGC 5*′*
10	dC: 5*′* TCCTACCGTGCCTACCTGAACAGCTGGTCATAGTCATGCCTACGAGTACG 3*′*
	3*′* TACGGATGCTCATGC 5*′*
11	dA: 5*′* TCCTACCGTGCCTACCTGAACAGCTGGTCTCGCTAATGCCTACGAGTACG 3*′*
	3*′* TACGGATGCTCATGC 5*′*
12	dG*′*: 5*′* TCCTACCGTGCCTACCTGAACAGCTGGTCTCGCT**A**A T GCCTACGAGTACG 3*′*
	3*′ G*T_s_A_s_CGGATGCTCATGC 5*′*
13	dOG: 5*′* TCCTACCGTGCCTACCTGAACAGCTGGTCTCGCT**A**A T GCCTACGAGTACG 3*′*
	3′*X*T_s_A_s_CGGATGCTCATGC 5*′*
*X* = *8oxodGMP*	

The template (1–4) and primer (5–7) oligonucleotides used and their corresponding DNA duplexes (8–13) are displayed. "s" denotes the phosphorothioate linkage between the nucleotides. For each of the template-primer duplexes, the templating nucleotides are underlined. In the case of the DNA duplexes utilized to asses exonuclease activity, the nucleotide present at the 3′ end is highlighted in italics and the corresponding nucleotide on the template strand is show in bold.

### Proofreading activity can remove 8oxodGMP misincorporated opposite template dA

**Figure 2:**
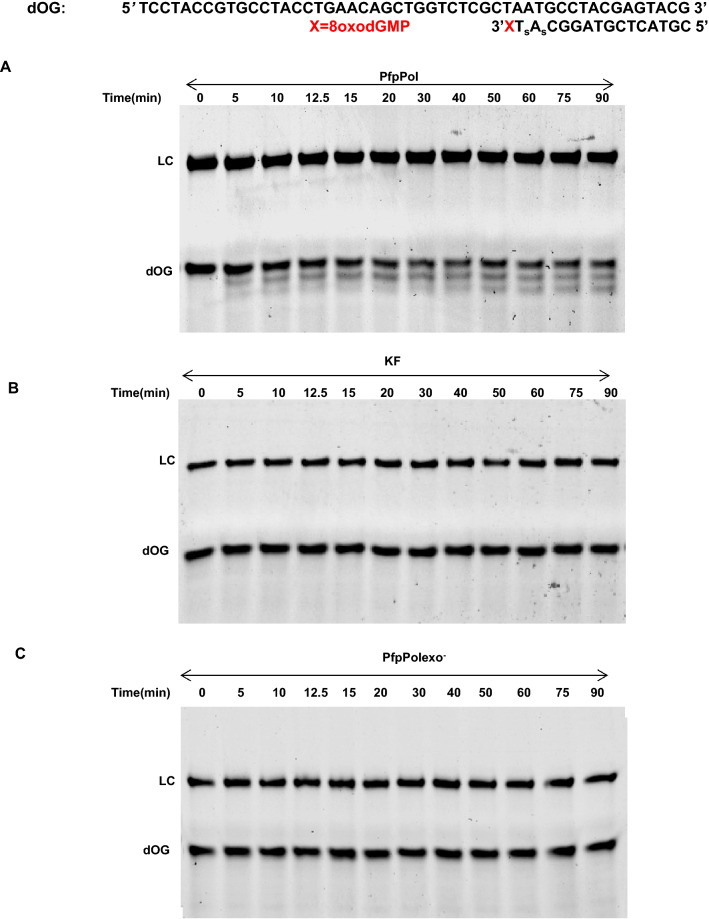
Excision activity on substrate bearing 8oxodGMP at the 3′ end. (**A**) 10 nM of PfpPol was incubated with 100 nM of dOG DNA substrate without dNTPs for different time points (0 to 90 min).The exonuclease activity of PfpPol can excise out 8oxodGMP present at the 3′ end of the primer opposite template dA. (**B**) 10 nM of KF was incubated with 100 nM of dOG DNA substrate without dNTPs for different time points (0 to 90 min). Unlike PfpPol, the KF enzyme is unable to excise the oxidized nucleotide present at the 3′ end of the primer. LC and dOG denote loading control and the DNA substrate with 8oxodGMP at the 3′ end, respectively. (**C**) PfpPolexo^−^ was used as a control, and as expected the exonuclease deficient version does not excise 8oxodGMP present at the 3′ end.

As an A-family dPol, PfpPol in addition to 5′-3′ polymerase activity, has a 3′-5′ exonuclease activity. The polymerase incorporates 8oxodGTP opposite dA with high frequency and can extend past this mismatch also. As a result, the mismatch can get fixed in the apicoplast genome and lead to transversion mutations after another round of replication. It is possible that the proofreading activity removes this error and therefore, the effect of the 3′-5′ exonuclease activity on the misincorporated 8oxodGTP was assessed, and it was observed that 8oxodGMP was successfully removed by PfpPol (Fig. 2A). KF is one of the best-studied A-family dPols and is also the closest structural homolog of PfpPol^32^ . The ability of the proofreading domain of the KF enzyme to remove 8oxodGMP was also evaluated. Even after a long incubation time of 90 min, the exonuclease activity of KF could not remove 8oxodGMP from the primer end (Fig. 2B). PfpPolexo^−^ was assayed as the control enzyme unable to remove 8oxodGMP from the primer end. (Fig. 2C). This observation suggests that the ability to remove misincorporated 8oxodGMP is not a general property of A-family dPols but is a unique attribute of the PfpPol enzyme. The proofreading ability, therefore, can reduce the frequency of 8oxodGTP induced transversion mutations in the AT-rich apicoplast genome of *Plasmodium falciparum*.

### Identification of residues that aid removal of 8oxodGTP

**Figure 3:**
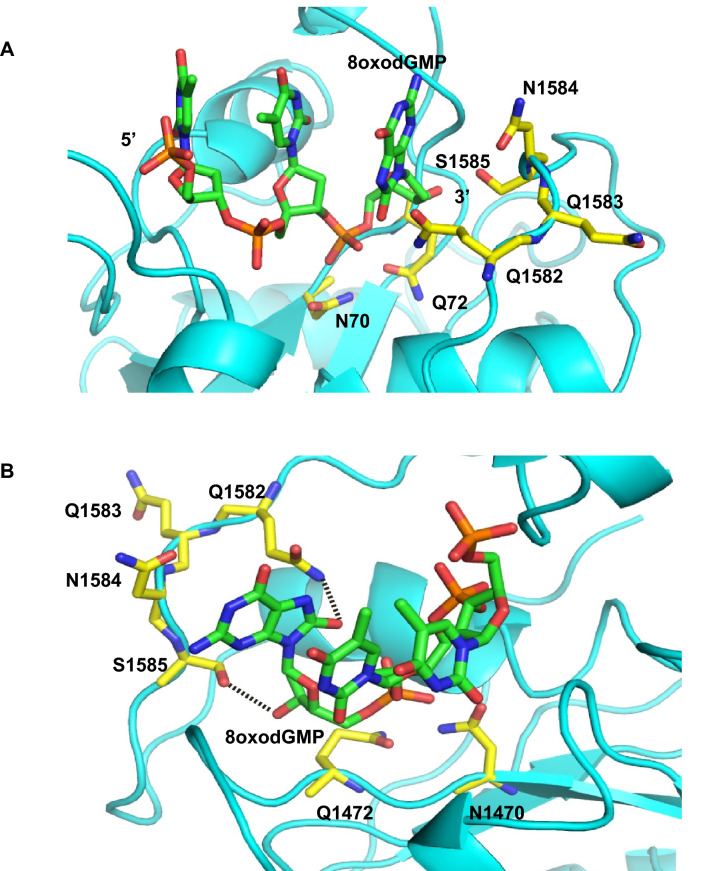
Model of the PfpPol exonuclease domain in complex with 8oxodGMP terminated single stranded DNA. (**A**) A computational model of PfpPol exonuclease domain in complex with DNA is displayed. The secondary structure elements and the residues are shown in cartoon and stick representation, respectively. The residues of the stretch ^1582^QQNS^1585^ were found close to 8oxodGMP. N1470 and Q1472 represent mutant versions of the catalytic residues, which are present close to the target phosphodiester bond. (**B**) The possible hydrogen bonds formed between S1585 and Q1582 with the 3′ end of the primer and the oxidized base, respectively, are displayed as dotted lines.

To identify the amino acid residues responsible for this unique activity of PfpPol, a computational model of the complex of the exonuclease domain with single-stranded DNA bearing 8oxodGMP at the 3′ end was prepared. For the computational model, the structure of the apo-PfpPol (5DKT) and the complex of KF with DNA (1KSP) were utilized. The model of the complex is composed of the exonuclease domain of PfpPol and a trinucleotide with 8oxodGMP present at the 3′ end (Fig. 3A). The energy of the complex converged to a minimum of − 14,515.3 kJ/mol after 3,000 steps of minimization. The energy-minimized model was analyzed to identify residues of the exonuclease domain that interact with 8oxodG residue at the 3′ end of the substrate DNA. The phosphodiester bond between 8oxodGMP at the 3′ end and the next nucleotide is located close to the catalytic residues. The model showed that the residues of the stretch ^1582^QQNS^1585^ in PfpPol interact with base moiety of 8oxodGMP to stabilize the damaged nucleotide in the exonuclease active site (Fig. 3A,B). Since these residues are unique to PfpPol, they might be responsible for the heightened exonuclease activity exhibited by PfpPol against 8oxodGMP. To test this hypothesis, the residues Q1582, ^1582^QQN^1584^, and S1585 were mutated to alanine by site-directed mutagenesis, and the corresponding proteins were purified to high homogeneity.

### Enhanced exonuclease activity of PfpPol enables excision of 8oxodGMP from primer terminus

**Figure 4:**
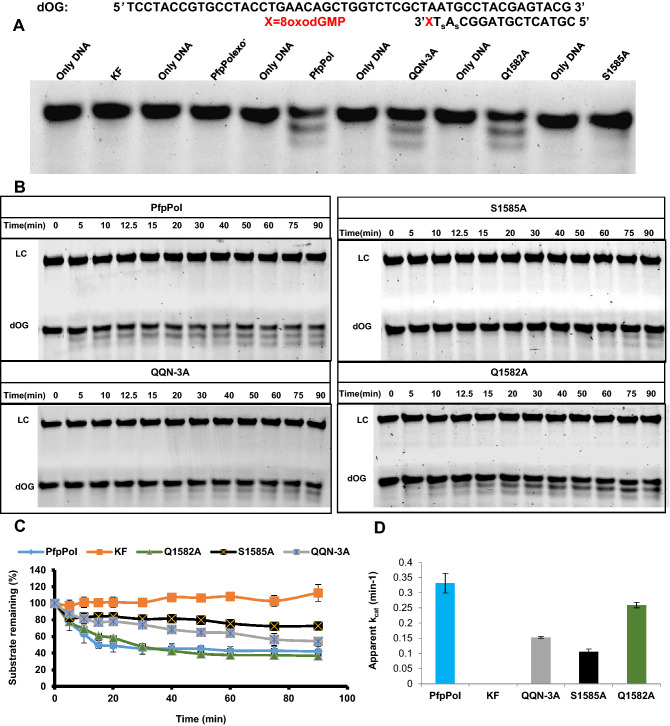
Proofreading activity of PfpPol and the mutant enzymes on dOG DNA substrate. (**A**) 10 nM of protein was incubated with 100 nM DNA (dOG) substrate without dNTPs for 1 h at 37 °C. PfpPol is able to remove 8oxodGMP from the primer end, in comparison the KF shows no excision activity. The PfpPolexo^−^ enzyme was used as a negative control. (**B**) Denaturing PAGE gels showing excision of 8oxodGMP from 0 to 90 min. LC and dOG denote loading control and the DNA substrate with 8oxodGMP at the 3′ end, respectively. Protein to DNA ratio was maintained at 1:10. S1585A and QQN-3A show reduction in their exonuclease activity as compared to PfpPol. (**C**) Percentage of the substrate (dOG) remaining plotted against time of incubation (0′ to 90′) for PfpPol, KF, QQN-3A, Q1582A and S1585A.The error bars denote standard deviation values (n = 3). (**D**) The rate of excision of 8oxodGMP was determined by incubating each of the proteins (10 nM) with DNA (100 nM) for 0 min and 15 min. In comparison to PfpPol, QQN-3A and S1585A showed two and three-fold reduction in excision activity, respectively.

The proteins PfpPol, KF, QQN-3A, Q1582A and S1585A were checked for their ability to remove 8oxodGMP from the primer end (Fig. 4A). The triple mutant QQN-3A showed a substantial decrease in the ability to remove 8oxodGMP from the 3′ end of the primer, and this observation highlights the importance of these unique residues of PfpPol (Fig. 4B). In the model, S1585 forms interactions with the 3′ –OH of the terminal primer nucleotide and therefore, may stabilize the terminal nucleotide in the correct orientation for productive catalysis (Fig. 3B). In line with this observation, the mutant protein S1585A showed more than a threefold reduction in excision activity as compared to wt-enzyme (Fig. 4C,D). Q1582 residue interacts with the oxidized base, but the mutant protein Q1582A exhibited only about 20% reduction in activity as the wt-enzyme (Figs. 3B, 4D). This may be because the flexibility in the unstructured polypeptide backbone in this region allows Q1583 to substitute for Q1582 (Fig. 3B). Overall, the analysis shows that the triple mutant and S1585A exhibit a twofold and threefold reduction in the ability to excise out 8oxodGMP while that of Q1582A mutant decreases by about 20% compared to wt- enzyme (Fig. 4C,D).

**Figure 5:**
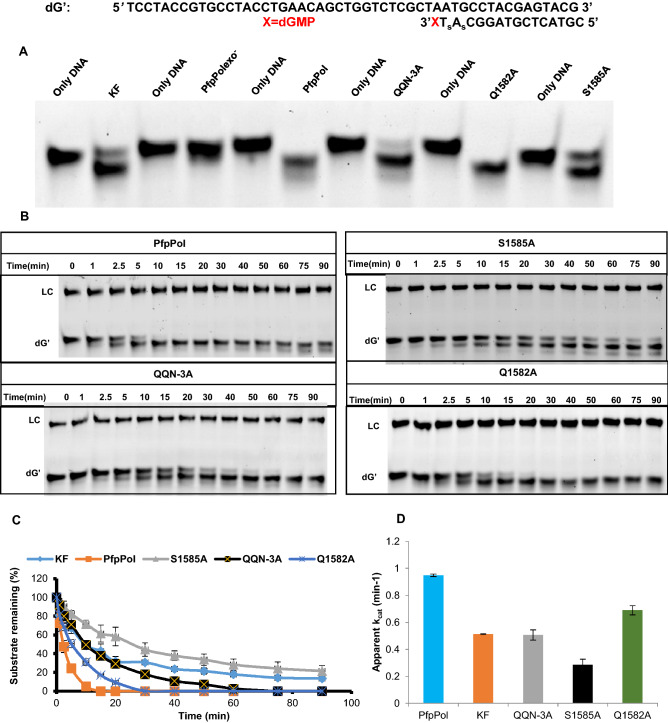
Proofreading activity of PfpPol and the mutant enzymes on dG′ DNA substrate. (**A**) 10 nM of each of the protein was incubated with 100 nM DNA (dG′) substrate without dNTPs for 1 h at 37 °C. S1585A and QQN-3A show reduction in their exonuclease activity on dGMP terminated primers as compared to PfpPol. PfpPolexo^−^ enzyme was used as a negative control. (**B**) Denaturing PAGE gels showing excision of dGMP from 0 to 90 min. LC and dG’ denote loading control and the DNA substrate with dGMP at the 3′ end, respectively. Protein to DNA ratio was maintained at 1:10. (**C**) Percentage of the substrate (dG′) remaining plotted against time of incubation (0′ to 90′) for PfpPol, KF, QQN-3A, Q1582A and S1585A.The error bars denote standard deviation values (n = 3). (**D**) Exonuclease rate of excision of dGMP was determined by incubating each of the protein (10 nM) with DNA (100 nM) for 0 min and 10 min. In comparison to PfpPol, QQN-3A and S1585A showed two and three-fold reduction in excision activity, respectively.

The exonuclease activity of the wt- and mutant PfpPol along with KF was also checked on mismatched DNA template primer duplex (dG') with a dA: dG mismatch at the primer 3′ terminus (Table 1). The ability of wt- and mutant versions of PfpPol to excise out dGMP from the primer terminus was assessed (Fig. 5A,B ). A comparison of the rate of reaction showed that there was a reduction in the activity of S1585A (~ threefold) and QQN3A mutant (twofold) as compared to PfpPol (Fig. 5C,D). The Q1582A mutant also showed a 27% reduction in excision activity.

A comparison between PfpPol and KF regarding the ability to process mismatched termini showed the activity of PfpPol is nearly double that of KF (Fig. 5D). These results suggest that the proofreading domain of the PfpPol enzyme is endowed with heightened exonuclease activity. As a result, the PfpPol enzyme has the unique ability to excise out oxidized nucleotides that have been misincorporated into the primer and thus prevent the appearance of ROS-induced transversion mutations.

**Figure 6:**
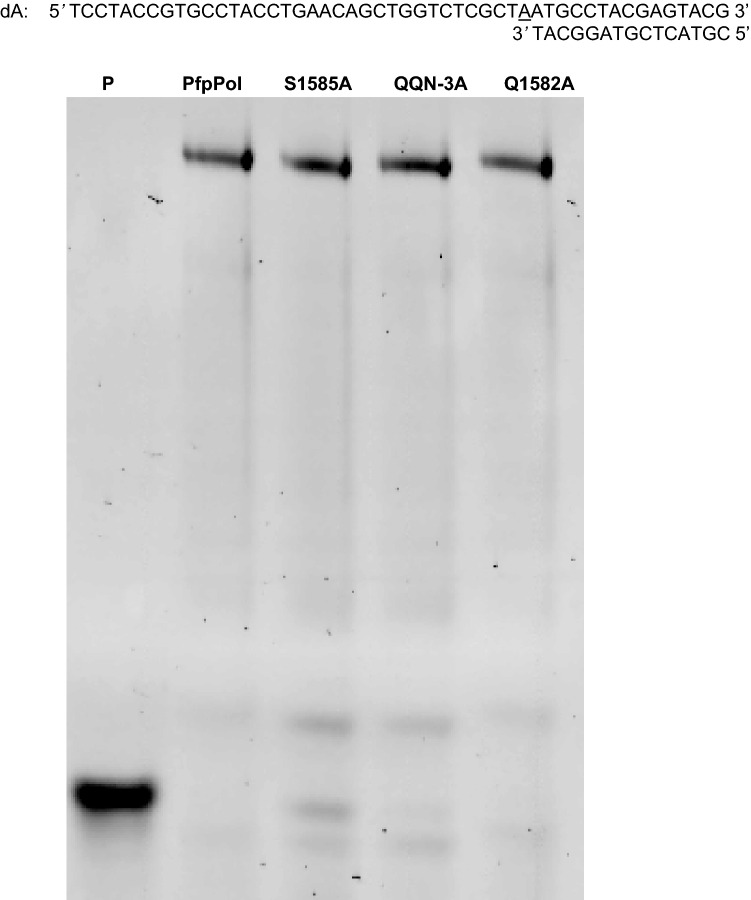
Primer extension activity. The DNA polymerase activity of S1585A, QQN-3A, and Q1582A were compared to that of PfpPol using primer extension assay. 20 nM of each of the protein was incubated with 100 nM of dA DNA substrate and 5 µM of dNTPs for 2 h at 37 °C and resolved on denaturing urea PAGE gel. S1585A, QQN-3A, and Q1582A were able to extend the primer to the same extent as PfpPol. P represents the DNA substrate alone. dA denotes the DNA substrate used in the experiment.

To check whether the polymerization ability of all the mutants (Q1582A, QQN-3A, and S1585A ) was intact, these proteins were incubated with substrate DNA (dA) and 5 µM of dNTPs. It was observed that all three mutant proteins (Q1582A, QQN-3A, and S1585A) were able to carry out 5′ to 3′ polymerase activity similar to wt-PfpPol (Fig. 6). The mutations in the exonuclease domain do not have any effect on the DNA polymerase activity of PfpPol.

## Discussion

**Figure 7:**
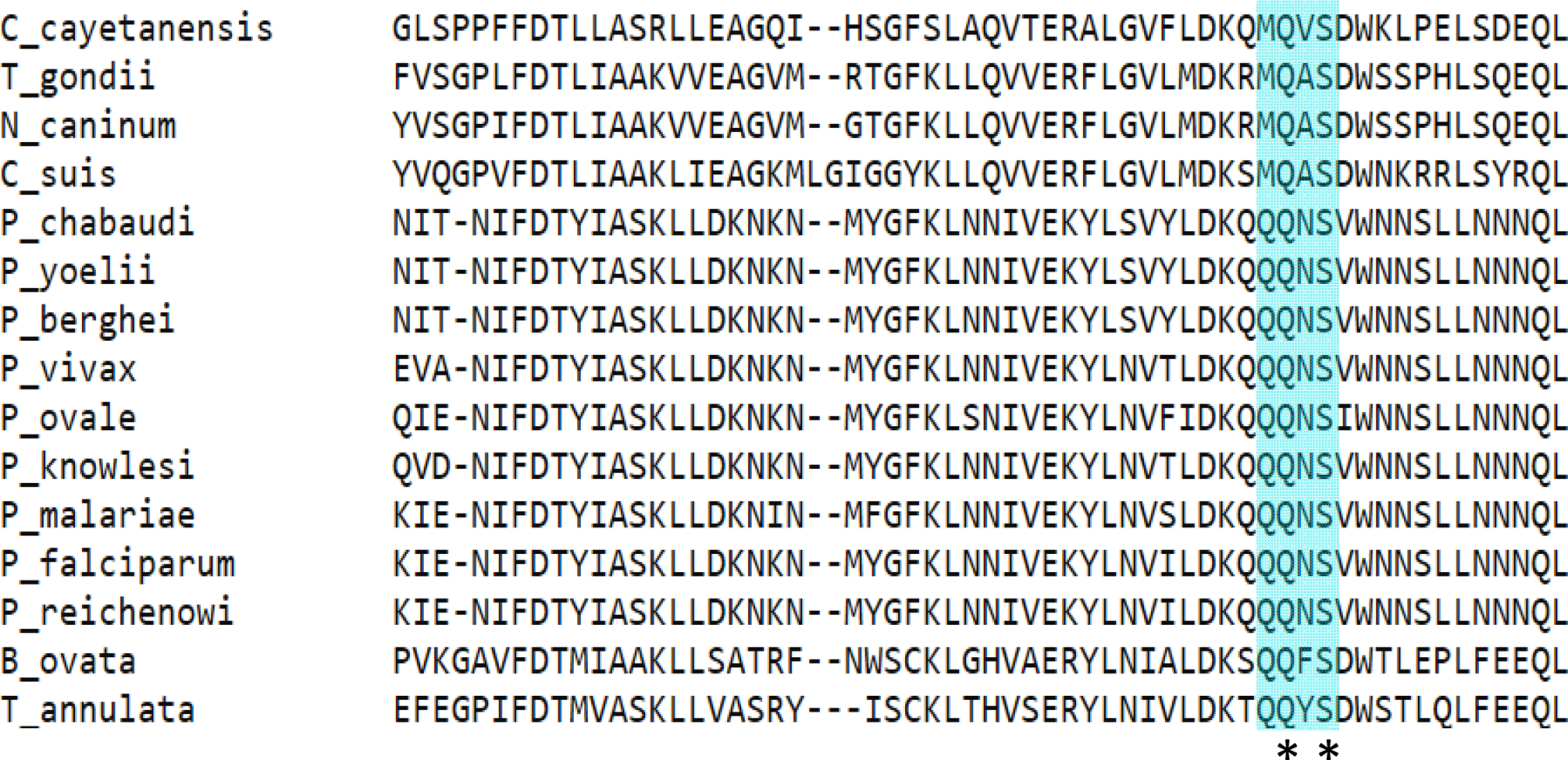
Sequence alignment of DNA Polymerases from different members of apicomplexa. The stretch ^1582^QQNS^1585^ is highlighted in cyan and the residues marked with an asterisk are conserved across different members of the apicomplexans. The QQNS motif is conserved across different members of the *Plasmodium* genera. The second Q and the S residues are conserved in many apicomplexans.

The A-family polymerases such as human Polymerase γ and T7 Polymerase exhibit inefficient excision of 8oxodGMP^21,33^ . T7 polymerase poorly excises out 8oxodGMP base-paired to template dA; however, it is efficient in removing dG incorrectly paired to dA^33^. Polymerase γ has also been shown to be more effective in extending the incorporated 8oxodGMP base-paired to dA rather than excising the mispair^3,21^. In our study, 8oxodGMP terminated primers were successfully excised by PfpPol and not by KF of DNA polymerase I (*E. coli*), another A-family polymerase with 3′-5′ exonuclease activity. The amino acid residues responsible for the exonuclease activity, Asp and Glu, are conserved in PfpPol and KF^32,34^. PfpPol possesses a unique stretch of amino acids in the exonuclease domain (QQNS) that substantially increases the proofreading ability of the exonuclease domain and this enhancement enables the enzyme to excise out oxidized nucleotides misincorporated into the primer. This stretch of residues is conserved in orthologues present in other members of the *Plasmodium* genera (Fig. 7). The second Q and the serine residue are conserved in many apicomplexans and therefore, the enhanced proofreading capacity and the ability to remove oxidized nucleotides may be a general feature of the plastidic dPol present in these organisms. Also, the polymerase activity of the PfpPol enzyme has the ability to prevent the incorporation of 8oxodGTP opposite dC. Therefore, the polymerase and proofreading activities of the enzyme act in concert to ensure that the damaged nucleotide is not added to the genome, neither opposite dC nor dA.

The nucleotide pool in the apicoplast is under constant oxidative stress as it is exposed to reactive oxygen species (ROS) produced during various metabolic processes that occur in this unique organelle^14,35,36^. The presence of ROS will lead to the generation of oxidized nucleotides such as 8oxodGTP that can lead to transversion mutations in the apicoplast genome^24,35–37^. In the mitochondria, even trace amounts of 8oxodGTP can substantially reduce the fidelity of replication of the mitochondrial genome^3^. Since the apicoplast genome of *P. falciparum* is highly AT-rich (~ 87%), the probability and frequency of 8oxodGTP: dA misincorporations will be high and therefore, the apicoplast genome is particularly vulnerable to mutagenesis by 8oxodGTP^38^. The apicoplast genome is known to code for about 30 genes and mutations in the apicoplast genome will lead to the altered proteins and RNA that cannot function properly and also lead to dysregulation of gene expression^39^. Overall, due to persistent oxidative stress, the AT-rich genome in the apicoplast is under constant threat and the unique attributes of the exonuclease domain of Pfprex may play an important role in protecting the apicoplast genome from the deleterious effects of ROS.

Previously, it has been shown that the Base excision repair, Mismatch repair, and Nucleotide excision repair pathways can detect and remove 8oxodGMP^40–44^. The present study shows that in the apicoplast, misincorporated 8oxodGMP present opposite template dA may be removed primarily by the proofreading activity of the replicative DNA polymerase. This property represents a new form of DNA repair wherein the proofreading activity resident in the DNA polymerase is the first enzyme to excise out promutagenic damaged nucleotides from the primer strand. The removal of damaged nucleotides is generally post-DNA synthesis^45^ and involves unloading of the DNA polymerase from the primer-template junction site. The observed activity of the exonuclease domain of Pfprex represents the first example of real-time dynamic error correction of damaged nucleotides wherein repair is concomittant with replication.

The perturbation of metabolic activities in the apicoplast has been shown to lead to a delayed death phenotype in apicomplexans such as *Plasmodium* and *Toxoplasma*^10,16,46,47^. These organisms are responsible for diseases in humans and the former is responsible for malaria, which causes a large number of fatalities in the tropical regions of the world^48^. According to WHO, there were approximately 4,05,000 deaths worldwide due to malaria in 2018, with nearly 85% of the fatal cases reported from 20 countries belonging to African and Indian region^49^. Despite the number of efforts to eradicate the disease, the mortality rate has not declined as expected, with nearly 435,000 deaths in 2017 and 451,000 in 2016^50^. This is partly because of the increased resistance to available drugs, including the present frontline artemisinin-based combination therapy^51–54^. This calls for an urgent need to devise better strategies and find new targets for drug development. The inhibition of the exonuclease activity of Pfprex may lead to deleterious transversion mutations in the apicoplast genome, which will decrease the overall viability of the parasite. The proofreading domain of Pfprex bears low homology to that of the only A-family dPols in humans with a functional proofreading activity such as Polγ. Hence, the exonuclease activity of Pfprex represents an attractive target for therapeutic intervention. The inhibitors of the proofreading activity may serve as effective adjuvants that potentiate the antimalarial activity of available therapeutics.

## Materials and methods

### Cloning, expression, and purification

The codon-optimized gene construct corresponding to Pfprex (Uniprot ID: Q8ILY1_PLAF7) was obtained from Genscript Inc and the gene segment corresponding to PfpPol (1,361–2016 aa residues, supplementary figure S1A) was cloned into the pGEX-6P-1 vector. The gene construct corresponding to the Klenow fragment of DNA polymerase I ((Uniprot ID: DPO1_ECOLI) was amplified from *E. coli* genomic DNA and cloned into the pGEX-6P-1 vector. All the mutants, namely D1470N + E1472Q (PfpPolexo^−^)^14^, Q1582A, S1585A and triple mutant QQN-3A(Q1582A + Q1583A + N1584A) were generated using QuikChange Lightning Site-Directed Mutagenesis Kit from Agilent. The cloned genes and the mutants were transformed into freshly prepared competent cells of the C41(DE3) strain of *E. coli*.

The cells were grown in LB media containing 100 µg/ml of ampicillin at 37 °C and continuous shaking at 180 rpm till OD_600_ was 0.7. Induction was done with 0.3 mM IPTG and cells were further grown for 16 h at 18 °C. The cells were pelleted down by centrifugation and resuspended in lysis buffer (500 mM NaCl, 5% Glycerol, 100 mM phosphate buffer (pH-7.5), 5 mM β-Mercaptoethanol, 0.01% IGEPAL and 1 mM phenylmethylsulfonyl fluoride). The cell lysate was sonicated and centrifuged at 17,800*g* for 50 min to obtain clear supernatant, which was subjected to purification by GST affinity chromatography. Briefly, the GST-sepharose beads (GE Healthcare Inc.) were equilibrated with buffer A (500 mM NaCl, 5% glycerol, 50 mM phosphate buffer (pH-7.5), 2 mM dithiothreitol (DTT), and 0.01% IGEPAL). The supernatant was then loaded on to the column and washed with buffer A, followed by buffer B (1 M NaCl, 5% glycerol, 50 mM phosphate buffer (pH-7.5), 2 mM DTT, and 0.01% IGEPAL). Protein was eluted after on-column cleavage with *PreScission* protease, which cleaves the GST tag of the protein. The eluted protein was concentrated and further purified by gel filtration chromatography using a Superdex-200 column (GE Healthcare Inc.). The protein was finally eluted in buffer containing 25 mM HEPES (pH 7.5), 500 mM NaCl, 5% glycerol and 2 mM DTT, concentrated and flash frozen.

### Primer extension assays

Primer extension assays were performed by annealing 5′ 6-FAM labeled primer P_15_ with four different templates A_T_, T_T_, C_T_ and G_T_ giving rise to DNA substrates dA, dT, dC and dG having dA, dT, dC and dG respectively at the templating position (Table 1). 30 nM PfpPolexo^−^ and 5 µM of either 8oxodGTP or dGTP were added to the reaction mixture consisting of 100 nM DNA substrate (Table 1) with 25 mM Tris–HCl (pH-7.5), 0.1 mM (NH4)_2_SO4, 0.1 mg/ml BSA, 10 mM MgCl_2_, and 1 mM DTT. The reaction was carried out for 30 min at 37 °C and terminated with stop solution consisting of 80% formamide, 1 mg/mL Xylene C, 1 mg/mL bromophenol blue, and 20 mM EDTA. This was followed by incubation at 95 °C for 4 min and quickly transferring to ice for 10 min. The mixture was loaded onto a 20% polyacrylamide gel containing 8 M urea and 1× TBE. Visualization of the products was carried out by excitation of the 5′ 6-FAM label at 488 nM using Typhoon scanner (GE healthcare). The amount of primer extended was calculated using the equation^55^:

$${\text{Percentage}}\;{\text{of}}\;{\text{primer}}\;{\text{extended}} = \frac{{{\text{I}}_{{\text{E}}} }}{{{\text{I}}_{{\text{E}}} + {\text{I}}_{{\text{U}}} }} \times 100$$

where I_E_ is the intensity of the extended band (n + 1) and I_U_ is the intensity of the unextended band (n) in the same lane.

For steady-state enzyme kinetics, 5 nM of PfpPolexo^−^ and 1 µM of DNA substrate were used for reactions. The time point at which 20% of the primer has been extended was chosen and increasing concentrations of either 8oxodGTP, dGTP, or dTTP were used to carry out reactions. Using Lineweaver-Burke plot, apparent *K*_*m*_, *V*_*max*_ and the catalytic efficiencies were calculated (Fig. 1B) with the help of standard protocols^24,56,57^.

To check complete polymerization of DNA by PfpPol and the mutants (Q1582A, S1585A and QQN-3A), 100 nM of DNA substrate, dA (Table 1) was used along with 5 µM of all dNTPs in a reaction mix consisting of 25 mM Tris–HCl (pH-7.5), 0.1 mM (NH4)_2_SO4, 0.1 mg/ml BSA, 10 mM MgCl_2_, and 1 mM DTT. 20 nM of each of the protein was added and the reaction was stopped after 2 h at 37˚C by the method described above.

### Extension post misincorporation of 8oxodGTP

Primer extension after incorporation of 8oxodGTP at the 3′ primer terminus was checked by polymerization reactions on the dOG DNA substrate. dOG was made by annealing template A_T_ with primer P_OG_; P_OG_ has 8oxodGMP at the 3′ terminus (Table 1). For comparison, polymerization was also checked on dA DNA substrate that was made by annealing P_15_ to template A_T_. P_15_ has dTMP at the 3′ terminus. 10 nM of PfpPolexo^−^ and 100 nM of DNA substrate (dOG) were used in reaction mix containing 5 µM of all dNTPs, 0.1 mM Ammonium sulfate, 0.1 mg/ml BSA, 10 mM MgCl_2_ , 1× of 5× assay buffer consisting of 125 mM Tris–HCl (pH-7.5) and 5 mM DTT. After 30 min and 1 h, the reaction was stopped as described above and extended bands visualized by excitation at 488 nM on Typhoon scanner (GE healthcare).

### Proofreading activity

3′ to 5′ exonuclease activity of PfpPol, KF, Q1582A, S1585A and QQN-3A was checked on dOG DNA substrate. The dOG substrate has 8oxodGMP already present at the 3′ primer terminus with non-hydrolyzable phosphorothioate linkages instead of phosphodiester linkages present at the penultimate and antepenultimate bonds linkages. Phosphorothioate linkages, which have sulfur in place of one of the oxygen atoms of a phosphate, prevented the exonuclease activity from degrading the DNA base pairs following the misincorporated oxidized base owing to long incubation times with the protein. The reaction mixture contained 10 nM of the proteins, 100 nM of DNA substrate, 0.1 mM Ammonium sulfate, 0.1 mg/ml BSA, 10 mM MgCl_2_, 25 mM Tris–HCl (pH-7.5) and 1 mM DTT. The reaction was stopped after an incubation of 1 h. Similarly, exonuclease activity was checked on dG' substrate that has dGMP at the 3′ primer terminus. For quantitative analysis, the reaction mix was incubated for different time points till 90 min to generate curves with the percentage of substrate remaining plotted against time. Two time points; 0 and 10 min and 0 and 15 min were chosen to estimate reaction rates for excision of dGMP and 8oxodGMP, respectively, as has been described previously^14^. To avoid errors due to loading, 100 nM of loading control was used in the reaction.

The percentage of substrate remaining was calculated using the equation:

$${\text{Percentage}}\;{\text{of}}\;{\text{substrate}}\;{\text{remaining}} = \left( {{{{\text{S}}_{{\text{i}}} } \mathord{\left/ {\vphantom {{{\text{S}}_{{\text{i}}} } {{\text{S}}_{0} }}} \right. \kern-\nulldelimiterspace} {{\text{S}}_{0} }}} \right)*100$$

where S_i_ = I_SRi_/I_LCi_; I_SRi_ is the intensity of the undigested primer band at time point i and I_LCi_ is the intensity of the loading control band at time point i with i ranging from time point 0 min to 90 min. S_0_ = I_SR0_/I_LC0_; I_SR0_ is the intensity of the undigested primer at time point 0 min and I_LC0_ is the intensity of the loading control at time point 0 min.

### Modeling studies

The structure of the exonuclease domain of KF bound to DNA (1KSP)^58^ was processed to isolate the exonuclease domain bound to three nucleotides. The complex of the KF exonuclease domain in complex with DNA and the apo structure of the exonuclease domain of PfpPol (5DKT)^32,58^ were superimposed onto each other. The DNA substrate was transferred from KF to PfpPol and the terminal nucleotide at the 3′ end was changed to 8oxodGMP. The model generated in this manner was subjected to energy minimization in Discovery Studio (Discngine SAS) using CHARMM force field^59–61^. The modeled structure of the PfpPol-exonuclease domain: DNA complex was analyzed using the CONTACT program of the CCP4 suite^62^ and all figures were prepared using the PyMOL program (Schrodinger Inc.).

### Sequence analysis

The fasta sequences of orthologs of Pfprex from different apicomplexans were obtained from NCBI website^63^. The sequences corresponding to the following organisms were used: *P. vivax* (SCO69476.1), *P. reichenowi* (XP_012765178.2), *P. malariae* (SBT72451.1), *P. ovale* (SCP06297.1), *P. knowlesi* (XP_002260883.1), *P. yoelii* (ETB59497.1), *P. berghei* (XP_022714368.1), *P. chabaudi* (SCM11163.1), *B. ovata* (XP_028865888.1), *T. gondii* (ACN59873.1), *C. cayetanensis* (OEH77393.1), *C. suis* (PHJ24259.1), *T. annulata* (XP_954352.1) and *N. caninum* (CEL66763.1). All the obtained sequences were then aligned with the exonuclease domain of Pfprex using the multiple sequence alignment tool, Clustal Omega, available at the EMBL-EBI website^64^.

### Accession numbers

UNIPROT: Q8ILY1_PLAF7 (Pfprex) and DPO1_ECOLI (DNA polymerase I) PDB: 1KSP (DNA polymerase I) and 5DKT (Pfprex) were used to model DNA in the exonuclease domain of PfpPol.

## Supplementary information

Supplementary file1 (PDF 312 kb)
