# Supplementary material for: The proofreading activity of Pfprex from Plasmodium falciparum can prevent mutagenesis of the apicoplast genome by oxidized nucleotides
Source: Sci Rep. 2020 Jul 7;10:11157. doi: 10.1038/s41598-020-67853-2 (PMC7341739; doi:10.1038/s41598-020-67853-2)
Supplement: Supplementary file 1 — Supplementary file1 (PDF 312 kb) [file 41598_2020_67853_MOESM1_ESM.pdf]

The proofreading activity of Pfprex from *Plasmodium falciparum* can prevent mutagenesis of the apicoplast genome by oxidized nucleotides

Minakshi Sharma, Naveen Narayanan and Deepak T. Nair

Figure S1

A.

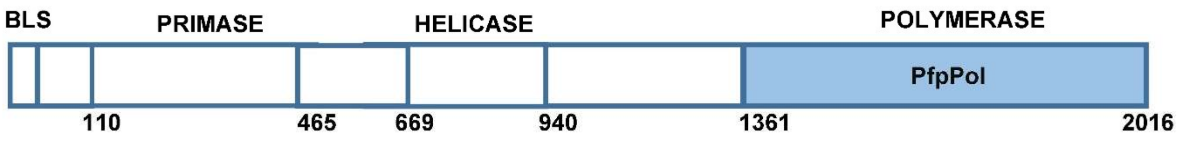

B.

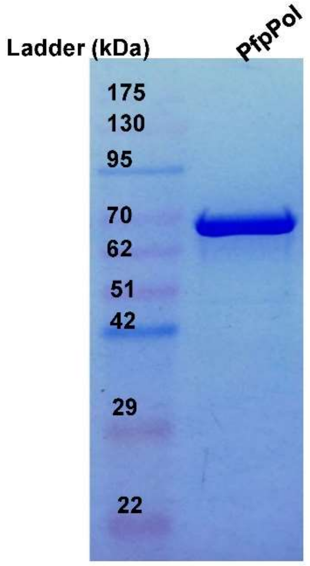

Fig .S1. Functional annotation of Pfprex and purification of PfpPol. (A) The PfpPol construct is shown in blue with numbers representing position of amino acid residues. BLS denotes the bipartite leader sequence present at the Nterminus. **(B)** SDS PAGE showing purified PfpPol along with a standard molecular weight marker (labelled in kDa).
